# Supplementary material for: EnzML: multi-label prediction of enzyme classes using InterPro signatures
Source: BMC Bioinformatics. 2012 Apr 25;13:61. doi: 10.1186/1471-2105-13-61 (PMC3483700; doi:10.1186/1471-2105-13-61)
Supplement: Addtional file 5 — The Java code to format the data files, evaluate and predict. The file enzml_java_code.tar.gz contains the Java code used to format database data to ARFF and XML formats, to execute cross and train-test (jackknife) evaluations and to record evaluation results to database. More information is included in the readme.txt file and the Javadoc files. The code can be used with a MySQL database. To use a different database software, other JDBC drivers might be required. [file 1471-2105-13-61-S5.gz › java_code/utils/doc/uk/ac/ed/inf/utils/database/DbManager.html]

DbManager


---


|  |  |  |
| --- | --- | --- |
| **Overview** \| **Package** \| **Class** \| **Use** \| **Tree** \| **Deprecated** \| **Index** \| **Help** | |  |
| **PREV CLASS**   **NEXT CLASS** | **FRAMES**    **NO FRAMES**     **All Classes** |  |
| SUMMARY: NESTED \| FIELD \| CONSTR \| METHOD | DETAIL: FIELD \| CONSTR \| METHOD |  |


---


## uk.ac.ed.inf.utils.database Class DbManager

```
java.lang.Object
  uk.ac.ed.inf.utils.database.Manager
      uk.ac.ed.inf.utils.database.DbManager
```

---

``` public class DbManager extends Manager ```

A database manager provides object to create a database, write and read from
it. A manager manages the trio: `DbCreator` to create the tables,
`DbReader` to read data from and `DbWriter` to write data to the
database.

**Version:**
:   20 Feb 2009

**Author:**
:   Luna De Ferrari luna.deferrari-at-ed.ac.uk

---

| **Field Summary** | |
| --- | --- |
| `DbWriter` | `m_dbWriter`             the database recorder |


**Constructor Summary**

`DbManager(java.lang.String dbConnPath)`
  
          Constructor 1: creates the database connection and the classes for
reading/writing to database


| **Method Summary** | |
| --- | --- |
| `void` | `closeConnection()` |
| `boolean` | `connectionIsValid()` |
| `java.lang.String` | `getDatabaseName()` |
| `DbConn` | `getDbConnection()` |
| `DbCreator` | `getDbCreator()` |
| `DbReader<java.lang.String,java.lang.String>` | `getDbReader()` |
| `DbWriter` | `getDbWriter()` |
| `TableManager` | `getTableManager(java.lang.String tableName)` |
| `TableManager` | `getTableManager(Table table)` |
| `void` | `refreshConnection()` |
| `void` | `runAnalyzeTable()`             Runs the 'ANALYZE TABLE tablename' SQL command for all the tables in a mysql database. |

**Methods inherited from class java.lang.Object**

`equals, getClass, hashCode, notify, notifyAll, toString, wait, wait, wait`

**Field Detail**

### m\_dbWriter

```
public DbWriter m_dbWriter
```

:   the database recorder


**Constructor Detail**

### DbManager

```
public DbManager(java.lang.String dbConnPath)
```

:   Constructor 1: creates the database connection and the classes for
    reading/writing to database


**Method Detail**

### closeConnection

```
public void closeConnection()
```

---


### connectionIsValid

```
public boolean connectionIsValid()
```

---


### getDatabaseName

```
public java.lang.String getDatabaseName()
```

---


### getDbConnection

```
public DbConn getDbConnection()
```

---


### getDbCreator

```
public DbCreator getDbCreator()
```

---


### getDbReader

```
public DbReader<java.lang.String,java.lang.String> getDbReader()
```

---


### getDbWriter

```
public DbWriter getDbWriter()
```

---


### getTableManager

```
public TableManager getTableManager(java.lang.String tableName)
```

---


### getTableManager

```
public TableManager getTableManager(Table table)
```

---


### refreshConnection

```
public void refreshConnection()
```

---


### runAnalyzeTable

```
public void runAnalyzeTable()
```

:   Runs the 'ANALYZE TABLE tablename' SQL command for all the tables in a
    mysql database. (Refresh indexes etc.) Good for performance.


---


|  |  |  |
| --- | --- | --- |
| **Overview** \| **Package** \| **Class** \| **Use** \| **Tree** \| **Deprecated** \| **Index** \| **Help** | |  |
| **PREV CLASS**   **NEXT CLASS** | **FRAMES**    **NO FRAMES**     **All Classes** |  |
| SUMMARY: NESTED \| FIELD \| CONSTR \| METHOD | DETAIL: FIELD \| CONSTR \| METHOD |  |


---
